# Key regulatory challenges in developing modified new chemical drugs in China: a national survey study

**DOI:** 10.3389/fphar.2025.1576013

**Published:** 2025-06-03

**Authors:** Lixia Fu, Guoshu Jia, Minji Wei, Yimin Cui

**Affiliations:** ^1^ Institute of Clinical Pharmacology, Peking University First Hospital, Beijing, China; ^2^ Beijing Key Laboratory of Clinical Pharmacology and Translation of Innovative Drugs, Beijing, China; ^3^ Department of Pharmacy Administration and Clinical Pharmacy, School of Pharmaceutical Sciences, Peking University, Beijing, China; ^4^ State Key Laboratory of Natural and Biomimetic Drugs, School of Pharmaceutical Sciences, Peking University, Beijing, China

**Keywords:** modified new drug, regulatory challenge, regulatory science, survey, clinical advantage

## Abstract

**Objective:**

With the increasing number of modified new chemical drugs (MNCDs) entering the market or in the approval pipeline in China, understanding their current status is crucial. This study aims to present stakeholders’ perspectives on the R&D and regulatory challenges associated with MNCDs.

**Methods:**

A nationwide online survey was conducted to collect perspectives from industrial stakeholders involved in drug R&D, drug manufacturing, and contract research organizations (CROs) and other related fields. A web-based questionnaire link was distributed to pharmaceutical professionals via the social media platform “WeChat”. Data were analyzed using descriptive statistics.

**Results:**

A total of 362 participants from 178 organizations across 19 provinces in China were recruited. Half of the respondents had over 10 years of work experience, 66% held intermediate or higher professional titles, and over 90% were experienced in R&D or regulatory submissions. Most respondents identified clinical advantage evaluation as the primary regulatory hurdle, with the lack of clear guidance and case references cited as significant impediments in the R&D and marketing application of MNCDs. Furthermore, clinical trial efficacy and safety data were identified as the main factors influencing the successful market launch of MNCDs, with expert consultation being the predominant method for assessing clinical advantages.

**Conclusion:**

Despite the issuance of several guidance for MNCDs, the industry still faces regulatory challenges in assessing the clinical advantages of these drugs. Additionally, there is a need for more objective and clear guidance in the R&D of modified new drugs. These findings are critical for regulators to refine the regulatory framework of MNCDs.

## 1 Introduction

Since China’s drug regulatory reform in 2015, a series of promotional policies have been implemented to encourage the development of new drugs, along with the readjustment of drug registration application classifications (Xu et al., 2018). The registration of chemical drugs are categorized into innovative drugs, modified new drugs, and generic drugs according to the *Provisions for Drug Registration* (State Administration for Market Regulation, 2020). The concept of modified new chemical drug (MNCD) was initially introduced in the National Medical Products Administration’s (NMPA) *Work Plan for the Reform of Chemical Drug Registration Classification* (China Food and Drug Administration, 2016) and later delineated in *Requirements for Registration Classification and Application Dossiers of Chemical Drugs* (National Medical Products Administration, 2020a). MNCDs that have not been marketed domestically or internationally refer to drugs whose structure, dosage form, formulation process, administration route or indications have been optimized based on known active ingredients, and that demonstrate significant clinical advantages (China Food and Drug Administration, 2016; National Medical Products Administration, 2020a). In China, the NMPA approves modified new drugs via the new drug application (NDA) pathway. Compared with innovative drugs and generic drugs, modified new drugs have become a research and development (R&D) hotspot in China. Similar to FDA’s non-NME (New Molecular Entity) classification, MNCDs represent drugs that retain the molecular features of NMEs but are often modified versions or combinations of already-approved pharmaceutical products. MNCDs are characterized by their low risk, short development cycle, high success rate, and high technical barriers, as they represent improvements on existing drugs (Hay et al., 2014). This has attracted more and more companies to invest in the R&D of MNCDs.

The registration classification of chemical drugs has undergone significant changes before and after the drug regulatory reform. In China, chemical drug products that are neither classified as innovative drugs nor generic drugs are defined as MNCDs. These MNCDs, which have not been previously marketed internationally, primarily apply for marketing authorization through the Class 2 registration pathway. The Class 2 registration pathway encompasses four types, as detailed in Table 1.

**TABLE 1:** The definition of Class 2 NDA registration pathway.

Classification code (NMPA)	Definition (National Medical Products Administration, 2020a)
Class 2	
Class 2.1	Drugs that contain an optical isomer of known active ingredients obtained by resolution or synthesis, or esterification of known active ingredients, or salification of known active ingredients (including salt containing hydrogen bonds or coordination bonds), or change in acid group, basic group, or metallic element of known active ingredients of salt, or formation of other non-covalent bond derivatives (e.g., complex, chelate or clathrate), and have significant clinical advantages
Class 2.2	Drugs that contain known active ingredients with new dosage form (including new drug delivery system), new formulation process or new route of administration, and have significant clinical advantages
Class 2.3	New compound preparations that contain known active ingredients and have significant clinical advantages
Class 2.4	Drugs for new indications that contain known active ingredients

For the review and approval of MNCD products, the NMPA has issued several documents, encompassing regulations and policies, as summarized in Table 2. The Center for Drug Evaluation (CDE), an affiliate to the NMPA, has also drafted several guidance documents related to the development and evaluation of MNCDs (Table 2). In December 2020, the CDE issued the *Technical Guidance for Clinical Trials of Modified New Chemical Drugs*. This guidance clarifies the principle for defining the clinical advantages of MNCDs and provides recommendations on how to demonstrate these advantages through clinical trials (National Medical Products Administration, 2020b). However, during the review process and in the course of communication and exchanges, drug reviewers at the CDE have received numerous inquiries from R&D companies and scientific research institutions. These questions primarily pertain to the interpretation of specific technical standards and review principles outlined in the guidance documents (National Medical Products Administration, 2022a). Subsequently, the CDE published a Question and Answer (Q&A) document related to the *Technical Guidance for Clinical Trials of Modified New Chemical Drugs* in 2022 to answer the common questions encountered during the development of MNCDs (National Medical Products Administration, 2022a). However, this Q&A document covers only eight specific questions, and it has not yet addressed the common issues encountered during the R&D process of MNCDS.

**TABLE 2:** Regulations, policies, and guidance documents related to MNCDs.

Document	Policy or Guidance name	Issuing authority	Draft/final	Date of Current version	Description	Reference
Regulation						
	Provisions for Drug Registration (Decree of the State Administration for Market Regulation No.27)	SAMR^a^	final	2020.01.22	The core document governing clinical trials and drug registration (Liu et al., 2022)	State Administration for Market Regulation (2020)
Policy						
	NMPA Issues Requirements for Registration Classification and Application Dossiers of Chemical Drugs	NMPA	final	2020.06.29	This document has issued guidelines outlining the registration classification and application dossier requirements for chemical drugs. The document clearly requires that modified new drugs should have clear clinical advantages	National Medical Products Administration (2020a)
Guidance						
	Technical Guidance for Clinical Trials of Modified New Chemical Drugs	CDE	final	2020.12.31	This document explains the clinical advantages of modified new chemical drugs, and how to prove their clinical advantages through clinical trials	National Medical Products Administration (2020b)
	Technical Guidance on Clinical Trials of Modified New Chemical Drugs for Children (Trial Implementation)	CDE	final	2021.09.13	Suggestions for clinical research of modified new drugs for pediatrics	National Medical Products Administration (2021)
	Technical Guidance on Clinical Pharmacokinetic Studies of Modified-Release New Drug Formulations	CDE	final	2022.01.07	This document aims to explain the general principles of design, implementation and evaluation of clinical pharmacokinetic studies of adjustable-release formulation in modified new drugs	National Medical Products Administration (2022b)
	Q&A on the Technical Guidance for Clinical Trials of Modified New Chemical Drugs (Draft for Comments)	CDE	draft	2022.03.14	This Q&A document compiled common issues encountered in the communication process with applicants regarding research and development of modified new drugs	National Medical Products Administration (2022a)
	Technical Guidance on Clinical Pharmacology Studies of Modified New Chemical Drugs (Trial Implementation)	CDE	final	2024.02.04	This document aims to provide guidance regarding the clinical pharmacology studies in the R &D of modified new drugs	National Medical Products Administration (2024)

^a^ SAMR, is short for State Administration for Market Regulation.

**FIGURE 1:**
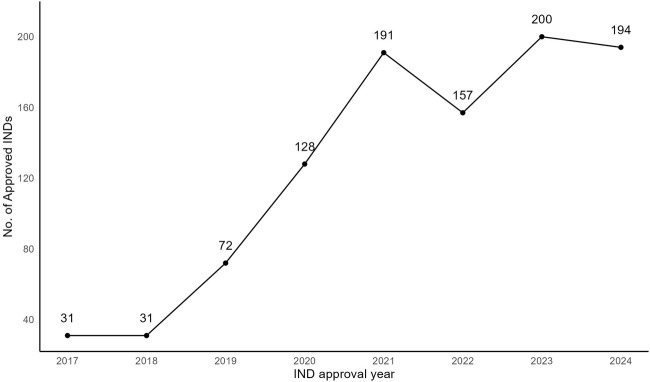
The number and trend of Class 2 MNCD INDs in China. The number of approved INDs in 2024 is only counted up to September.

As of September 2024, the number of MNCD INDs has increased gradually from 2017 to 2024, with different strengths of the same application being counted as a single IND [Figure 1, the data was extracted from the commercial database Pharmacodia (Pharmacodia, 2024)], and Table 3 shows the annual number of IND approvals for each category of Class 2.1-2.4. However, the guidance can only provide general navigation, there may still be a discrepancy in understanding the regulatory requirements between regulators and applicants, and this may add the hurdles to the R&D of MNCDs. Moreover, it remains unclear whether MNCDs face related regulatory issues and challenges during the R&D and market launch processes. In addition, the new chemical drug registration classification system has been implemented for 8 years. However, to our knowledge, there have been no surveys carried out in China to assess the current industry status and stakeholders’ perspectives regarding the modified new chemical drug regulations. Notably, this represents a significant gap in the existing literature.

**TABLE 3:** The number of approved MNCD INDs through Class 2 NDA pathway.

	No. of Approved INDs (n)										
Classification code	2.1	2.2	2.3	2.4	2.1; 2.4	2.2; 2.4	2.1; 2.2; 2.4	2.1; 2.2	2.2; 2.3	2.3; 2.4	2.2; 2.3; 2.4
Approval year											
2017	2	8	1	20	0	0	0	0	0	0	0
2018	4	19	0	5	1	2	0	0	0	0	0
2019	1	16	3	46	0	5	1	0	0	0	0
2020	4	39	7	71	0	5	0	1	1	0	0
2021	3	63	9	100	0	11	1	2	2	0	0
2022	5	69	10	62	1	9	0	1	0	0	0
2023	4	85	15	71	3	18	3	0	0	1	0
2024^a^	5	81	17	66	0	17	3	1	1	2	1

^a^ The number of approved INDs in 2024 is only counted up to September.

To improve the R&D and regulation of MNCDs, it is of great importance to identify and understand the current state of MNCDs. Therefore, from the perspective of regulatory science, we conducted a nationwide survey of relevant personnel involved in the field of modified new drug research, aiming to understand the key regulatory gaps and determine whether MNCDs is facing significant challenges in the development and regulatory approval process. Additionally, this study seeks to provide suggestions for exploring and developing new standards, tools, and methods for regulating MNCDs. The survey results may provide valuable decision-making support to policymakers, pharmaceutical companies, and regulatory agencies, facilitating further regulatory reform and the efficient review and regulation of MNCDs. Moreover, this study may offer recommendations and references to promote the translation of innovative research achievements, enhance clinical drug accessibility, and further optimize medications to provide patients with better treatments. In this paper, the term “modified new drugs” specifically refer to MNCDs as mentioned in the following text.

## 2 Methods

### 2.1 Questionnaire design and pilot survey

Firstly, we consulted six experts who are engaged in MNCDs area regarding the regulatory challenges currently faced in the R&D and marketing processes on a small scale. These experts are from the pharmaceutical industry, contract research organizations (CROs), and academic research institutions. Then, group members with a background in drug regulation formed a project team to design a pre-survey questionnaire for MNCDs. Furthermore, we conducted a pilot survey by sending this pre-survey questionnaire to twelve industry experts well-versed in modified new drugs to ensure the feasibility and reliability of the questionnaire, allowing for subsequent refinements and enhancements. Finally, the final version of the survey questionnaire was formulated in Chinese (the English questionnaire is included in the Supplementary file 1) and created via the Wenjuanxing website (https://www.wjx.cn/).

The cover letter of the survey questionnaire provides a concise overview, encompassing the study’s background, objective, and procedures, along with “confidentiality measures” and “information security confirmation.” The survey comprises forty questions with various data entry formats, including single-choice, multiple-choice, ranking options with open-ended comments, 5-point Likert scale items, and free-text responses.

The questionnaire was divided into four sections: general background information, key issues and challenges, regulatory challenges, and regulatory considerations. The first section consists of sixteen questions covering the general background information of the respondents, including gender, age, education level, professional title, work field, and years of experience, etc. The second section, titled “Key issues and challenges in the development and market launch of modified new drugs,” includes seven questions focusing on perspectives regarding key issues, challenges and considerations in the R&D, registration application and market launch process of MNCDs. The third section, titled “Regulatory challenges in the registration application of modified new drugs,” aims to explore and understand the regulatory issues and challenges encountered during the application and market launch process. The fourth section consists of nine questions on a 5-point Likert scale (Singleton et al., 2021), addressing the respondents’ understanding degree of regulatory policies and the need for regulatory improvements. Each question was followed by an option for respondents to provide open-ended comments. The final two questions of the survey were open-ended, allowing respondents to freely offer suggestions regarding the regulation of modified new drugs.

### 2.2 Participant recruitment and survey dissemination

Following the design of the questionnaire, an online access web link was created and mainly distributed to professionals within the pharmaceutical industry through the social media platform “WeChat”. This distribution was conducted over a period spanning from September 23rd to October 21st, 2024. WeChat (Weixin in Chinese) has become one of the most widely and frequently used social and professional mobile platforms in China (Pang, 2022). A combination of convenience and snowball sampling techniques was employed to recruit participants (Liu et al., 2024; Hu et al., 2022). Participants were not provided with any compensation for their involvement.

### 2.3 Data collection and statistical analysis

Data were collected and stored via Microsoft Excel version 2021 software (Microsoft Corp). For the ranking questions, the frequency of selection for each option was counted, and weighted scores were calculated based on the order of ranking (ranking 1st, 2nd and 3rd received 3, 2, and 1score, respectively). For the 5-point Likert scale, answers were converted to a numerical scale ranging from 1 point (strongly disagree/not at all, or equivalent wording) to 5 points (strongly agree/very likely, or equivalent wording). Mean scores and standard deviations (mean ± SD) were calculated to assess the relative contribution of each item to the total scale score. Overall, categorical variables were analyzed using descriptive statistics, while the 5-point Likert scale data were analyzed quantitatively. Responses to open-ended questions were organized into prevalent themes for analysis. Statistical analyses were conducted using Microsoft Excel version 2021 software (Microsoft Corp), R version 4.3.3 (R Project for Statistical Computing), and RStudio software version 2023.09.1 (RStudio, PBC).

## 3 Results

### 3.1 Characteristics of survey participants

A total of 365 responses were collected, of which 3 were identified as invalid and excluded due to being submitted by the same individual based on matching ID and IP address. Therefore, 362 valid responses were included in the final analysis. The demographic and professional characteristics of the participants are presented in Table 4.

**TABLE 4:** Characteristics of survey respondents.

Characteristics	Participants, n (%) (N = 362)
Gender	
Male	173 (48%)
Female	189 (52%)
Age Group	
<25	12 (3.3%)
25–34	138 (38%)
35–44	132 (36%)
45–54	45 (12%)
55–64	33 (9.1%)
>65	2 (0.6%)
Highest level of education	
Below undergraduate	5 (1.4%)
Undergraduate	120 (33%)
Master	176 (49%)
PhD	61 (17%)
Years of work experience	
Less than 1 year	20 (5.5%)
1–5 years	65 (18%)
5–10 years	92 (25%)
10–15 years	70 (19%)
Over 15 years	114 (31%)
Others	1 (0.3%)
Professional Title	
Senior	98 (27%)
Mid-Senior	53 (15%)
Intermediate	88 (24%)
Junior	103 (28%)
Others	20 (5.5%)
Province	
Guangdong	167 (46%)
Beijing	69 (19%)
Jiangsu	25 (6.9%)
Shanghai	23 (6.4%)
Zhejiang	17 (4.7%)
Sichuan	14 (3.9%)
Shandong	12 (3.3%)
Others^a^	35(9.8%)
Organization Nature	
Domestic-funded enterprise	268 (74%)
Foreign-funded enterprise	26 (7.2%)
Sino-foreign joint venture	22 (6.1%)
Public institution	35 (9.7%)
Others	11 (3.0%)
Developing modified new drugs	
Yes	308 (85%)
No	35 (9.7%)
Planning	19 (5.2%)
Highest R&D Stage	
Academic Research	22 (6.7%)
Project Initiation Phase	18 (5.5%)
Pharmaceutical Research	30 (9.2%)
Preclinical Research	22 (6.7%)
IND or Filing	19 (5.8%)
Phase 1 Clinical Study	37 (11%)
Phase 2 Clinical Study	27 (8.3%)
Phase 3 Clinical Study	33 (10%)
Marketing Application Phase	44 (13%)
Post-marketing Research	75 (23%)

Note.

^a^ Including the other 12 provinces.

The respondents’ gender distribution was approximately equal, with each gender accounting for about 50%. The majority of the questionnaire respondents were aged between 25 and 44 years old, making up 74% of the total. The remaining age groups were distributed as follows: 3.3% were under 25, 12% were aged 45–54, 9.1% were aged 55–64, and 0.6% were over 65. The vast majority respondents (98.6%) held at least a bachelor’s degree, with 66% possessing a master’s degree or higher. Regarding working experience, 50% of the respondents had more than 10 years of experience, and 66% held intermediate or higher professional titles. Notably, nearly a third of the highly experienced respondents had more than 15 years of work experience and senior professional titles. Most respondents (74%) were employed in domestic-funded enterprises, and 26% worked in other types of organizations, such as foreign-funded enterprises, public institutions, and so on.

**FIGURE 2:**
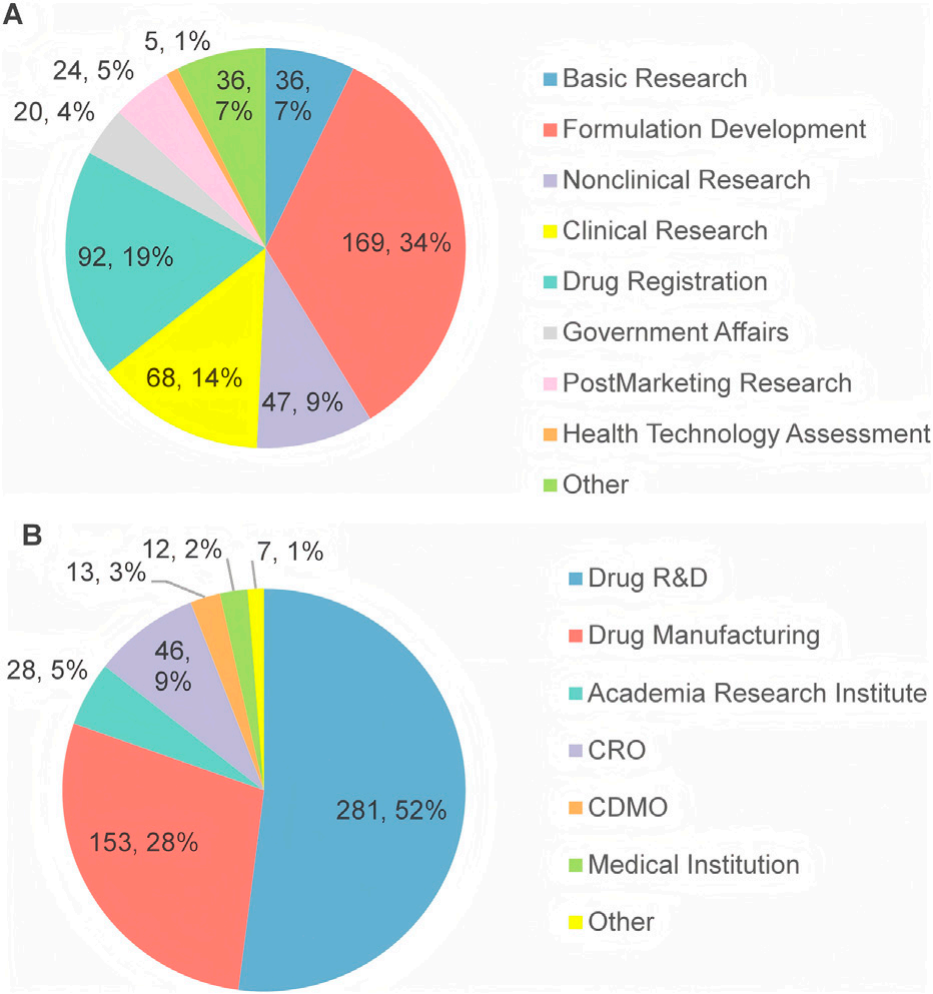
The work field **(A)** and the main domain **(B)** of the respondents’ affiliations.

All participants were affiliated with over 178 institutions across 19 provinces in China, with the largest proportion of respondents from Guangdong Province (46%), followed by Beijing (19%) and Jiangsu Province (6.9%). Regarding their academic and professional backgrounds, 34% of respondents are engaged in the field of pharmaceutical formulation, 19% in drug registration, 14% in clinical research, 9% in nonclinical research, and 7% in basic research (Figure 2A). As Figure 2B shows, 52% of the respondents were affiliated with drug R&D enterprises, and 28% with drug manufacturing enterprises.

**FIGURE 3:**
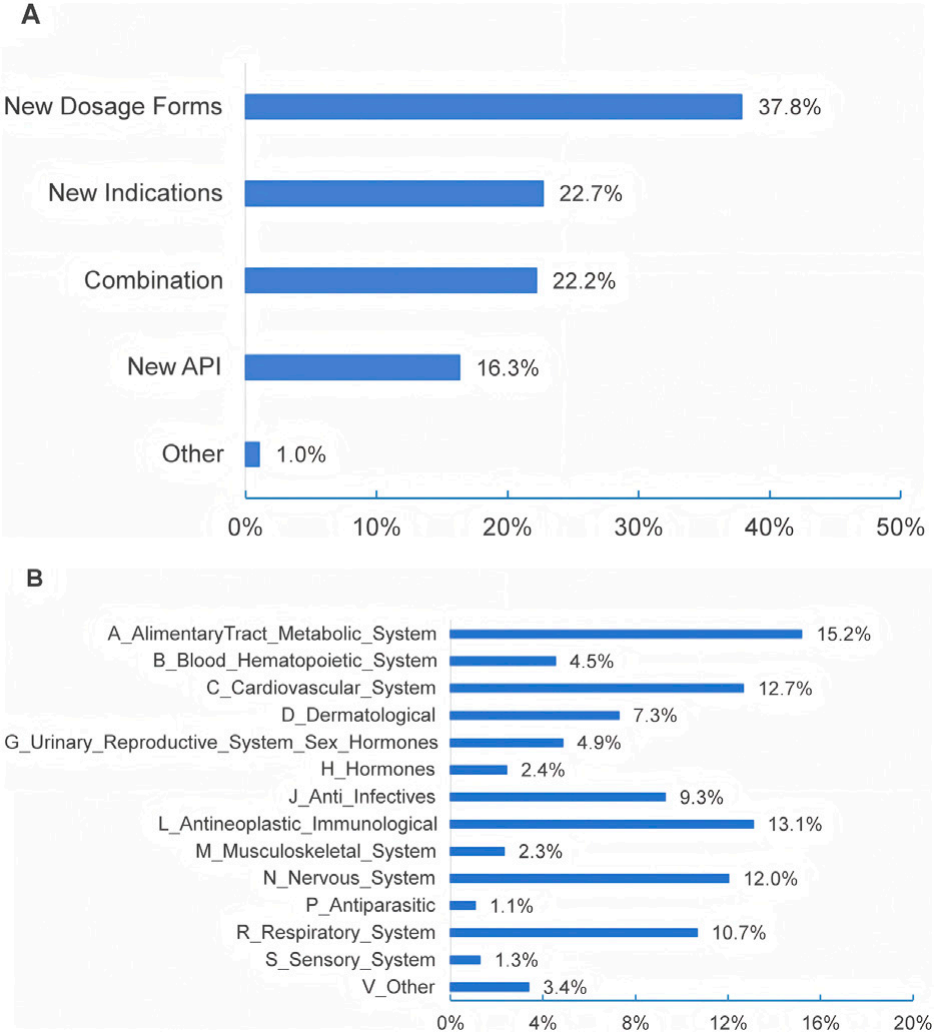
The types **(A)** and indications **(B)** of MNCDs that are being developed or followed.

Questions 14–16 in the first section were tailored for respondents based on their institutions’ involvement in the development of MNCDs, as specified in Question 13. As Table 4 shows, 85% of the respondents reported that their primary institutions are actively developing MNCDs, with 5.2% planning to engage in MNCDs development. The highest R&D stages of MNCDs in their organizations span all R&D phases (Table 4, ranging from basic research to post-marketing studies), MNCD types (Figure 3A), and therapeutic indications (Figure 3B).

### 3.2 Key issues and challenges in the R&D and market launch of MNCDs

**FIGURE 4:**
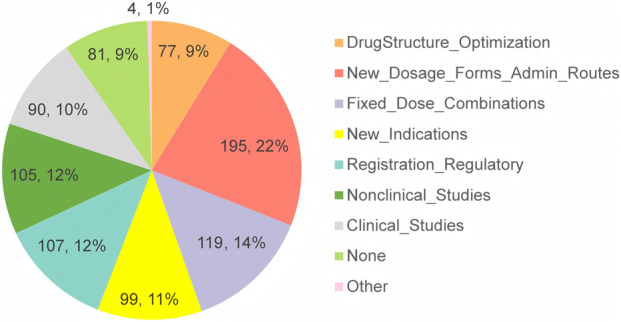
The respondents’ experience related to MNCDs.

The second section began with a question designed to assess the respondents’ experience in the R&D or registration application for MNCDs. As depicted in Figure 4, the vast majority of respondents (90.3%) reported having experience across various aspects of MNCDs R&D, while only 9.2% of the respondents indicated no experience in this area.

Furthermore, we investigated the key issues and critical challenges in the R&D and marketing authorization process of MNCDs. To gain insights into how stakeholders view the issues and challenges associated with the R&D and regulatory submission of MNCDs, the second section of this survey included the following ranking questions:

• What are the main issues you encounter during the drug registration and submission process?
• What do you consider the main challenges currently faced in the R&D and market application of modified new drugs?
• What factors do you think have the greatest impact on the successful market launch of modified new drugs?
• During the development phase of modified new products, what approach does your organization most commonly use to assess clinical advantages?

**FIGURE 5:**
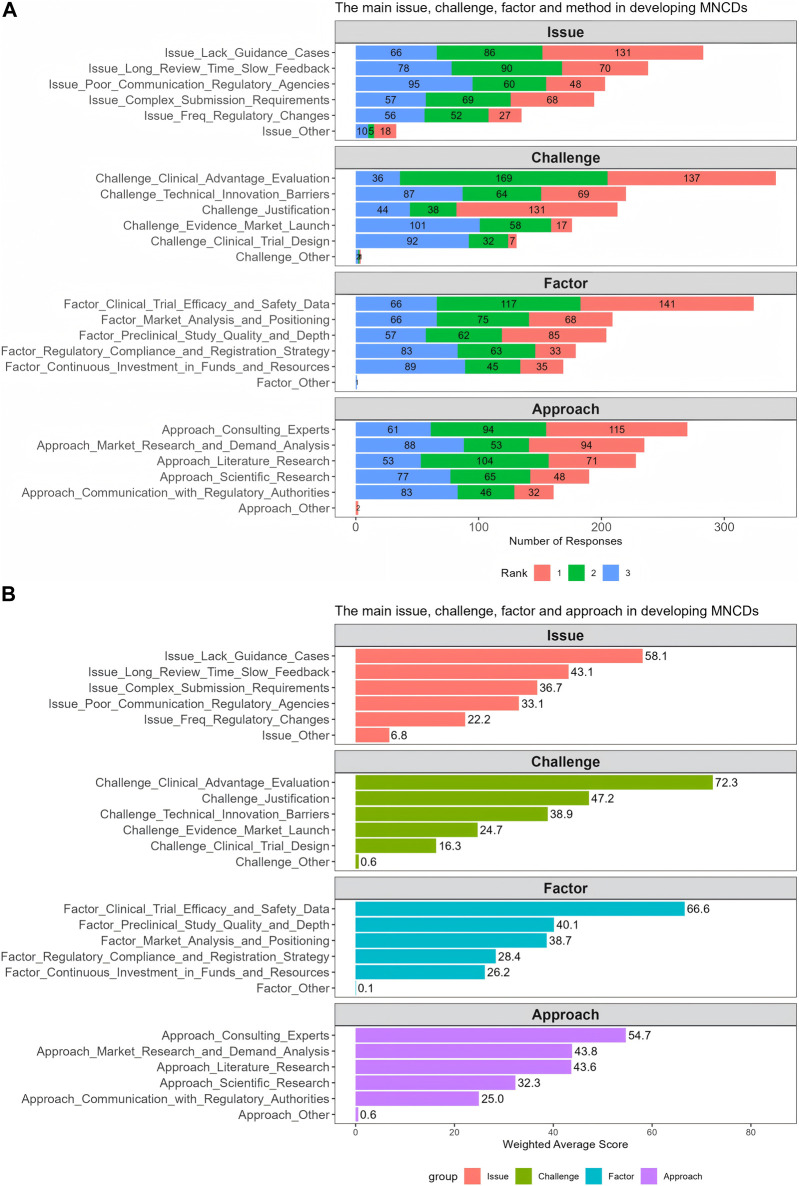
The frequency distribution **(A)** and weighted average score **(B)** of challenges, factors, issues and approaches for developing MNCDs.

The respondents were requested to prioritize the top 3 key issues, challenges, factors, and approaches for each of the four questions mentioned above. Figure 5A presents the results of frequency distribution of responses to the four ranking questions. Overall, most respondents perceived that the primary regulatory challenge faced in the R&D and regulatory submission process of MNCDs was assessing and demonstrating clinical advantages, while the main issue was a lack of clear guidance and case references (Figure 5A). Additionally, most respondents consider clinical trial efficacy and safety data as the main factor impacting the successful regulatory approval. The most common way they used to demonstrate clinical advantage was to consult experts.

Figure 5B presents the weighted average scores for these challenges, factors, issues, and approaches, with results similar to the frequency findings (Figure 5A). Compared to other challenges, the challenge of assessing and demonstrating clinical advantages received the highest score (72.3), followed by the basis for project initiation (47.2) and breaking through technical barriers and innovation (38.9). The factor that impacts the most was the clinical trial efficacy and safety data which scored 66.6, indicating a critical area needing attention. In addition, the findings have highlighted a lack of guidance as a significant issue and have identified expert consultation as a preferred approach among the various strategies considered. These insights reflect the prevailing issues and strategic approaches in the R&D and regulatory submission process of MNCDs.

**FIGURE 6:**
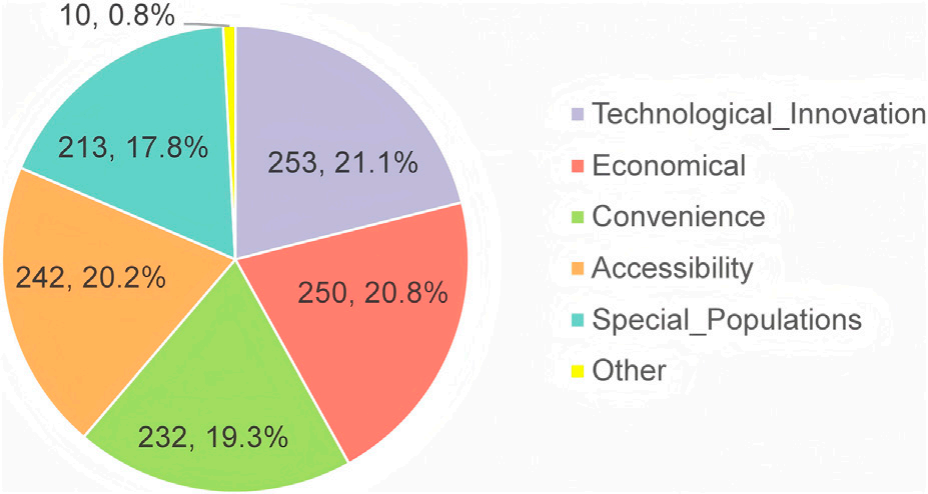
Other dimensions that may need to be considered.

The regulatory requirements for clinical advantage assessment refer to effectiveness, safety, and compliance of MNCDs (National Medical Products Administration, 2020b). However, over a half of the respondents believe those other dimensions also needed to be considered, including technological innovation, economic viability, convenience, accessibility, and special populations (Figure 6). Respondents were queried regarding their perspectives on the future prospects of MNCDs (Table 5). Despite facing strict regulatory challenges, opinions were divided: about half of the respondents believe that there are still substantial unmet clinical needs with broad prospects. In contrast, the other half perceive that there are specific clinical needs, with a moderate market size. Overall, the majority of respondents maintain an optimistic outlook regarding the future prospects of MNCDs.

**TABLE 5:** Opinions on the prospects of MNCDs.

Opinions on Modified New Product Prospects	Participants, n (%^a^) (N = 362)
There is still a large amount of unmet clinical needs, with broad prospects	168 (46%)
There are specific clinical needs, with a certain market space	181 (50%)
Clinical needs are basically met, and the market is close to saturation	3 (0.8%)
Uncertain	10 (2.8%)

^a^ Sums may not total to 100% because of rounding.

### 3.3 Regulatory challenges in the registration application of MNCDs

The third section explored the respondents’ opinions on regulatory issues encountered during the regulatory submission process of MNCDs. The survey posed the following questions:

• Which policies or regulations do you think have the greatest impact on the R&D of MNCDs?
• What do you think is the most important focus in the regulatory review process for modified new drugs?
• What do you think is the most important area that regulatory agencies need to improve in the review process of modified new drugs?

As illustrated in Table 6, 45% of the participants identified “drug registration and review policies” as the most influential factor affecting the R&D of MNCDs. Additionally, more than half (69%) of the respondents considered the clinical advantage of MNCDs to be the greatest concern during the review process for these drugs. When inquired about the aspects that most in need of improvement in the process of reviewing, respondents selected different options: 30% thought that the transparency and consistency of review criteria should be enhanced, followed by 28% suggested providing more guidance and case references. Furthermore, 19% prioritized improving communication and feedback mechanisms with enterprises, 12% emphasized strengthening the professional training and capacity building of reviewers, and 8.8% opted to optimize review processes and time management.

**TABLE 6:** Participants’ response to the regulatory challenges related to MNCDs.

Question	Opinion, n (%^a^) (N = 362)
Policies or Regulations Impacting Modified New Drug R&D	
Drug registration and review policies	163 (45%)
Regulations related to clinical trials	54 (15%)
Intellectual property protection and data exclusivity	72 (20%)
Drug safety and quality standards	23 (6.4%)
Market access and healthcare insurance policies	49 (14%)
Others	1 (0.3%)
Most Important Concerns in Modified New Drug Review	
Clinical advantage	248 (69%)
Clinical evidence	76 (21%)
Non-clinical evidence	3 (0.8%)
Formulation process	8 (2.2%)
Quality control	23 (6.4%)
Others	4 (1.1%)
Most Needed Improvement in Regulatory Review Process	
Enhancing the transparency and consistency of review criteria	110 (30%)
Strengthening professional training and capacity building of reviewers	45 (12%)
Optimizing review processes and time management	32 (8.8%)
Improving communication and feedback mechanisms with enterprise	68 (19%)
Providing more guidelines and case references	103 (28%)
Others	4 (1.1%)

^a^ Sums may not total to 100% because of rounding.

**FIGURE 7:**
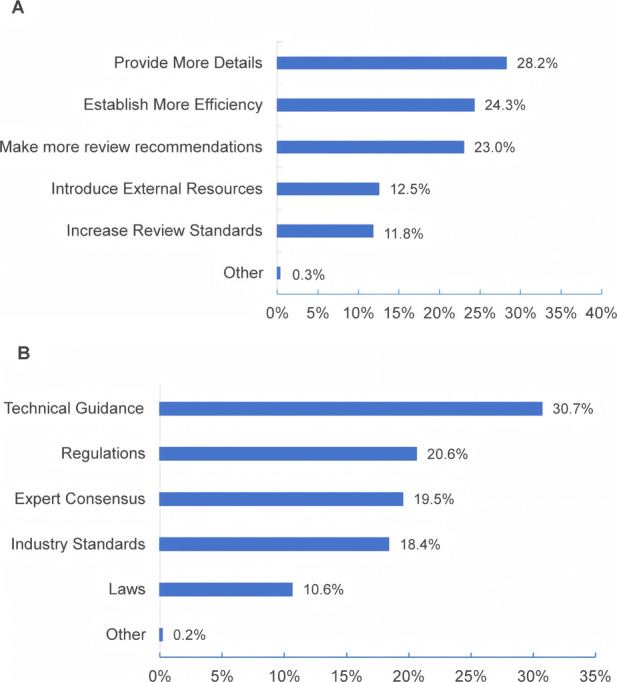
Suggestions and aspects to optimize for the regulation of MNCDs.

Regarding suggestions for enhancing the transparency and efficiency of the review process for modified new products, Figure 7 illustrates that over 20% of respondents selected “provide more detailed review feedback and guidance” (28.2%), “establish more efficient communication channels” (24.3%), and “make more review report information public” (23.0%). For the construction and refinement of regulatory systems and technical standards for modified new drug products, approximately a third (30.7%) chose technical guidance, followed by regulations (20.6%). In addition, 19.5% also favored expert consensus, and 18.4% chose industry standards.

### 3.4 Regulatory considerations and suggestions

**FIGURE 8:**
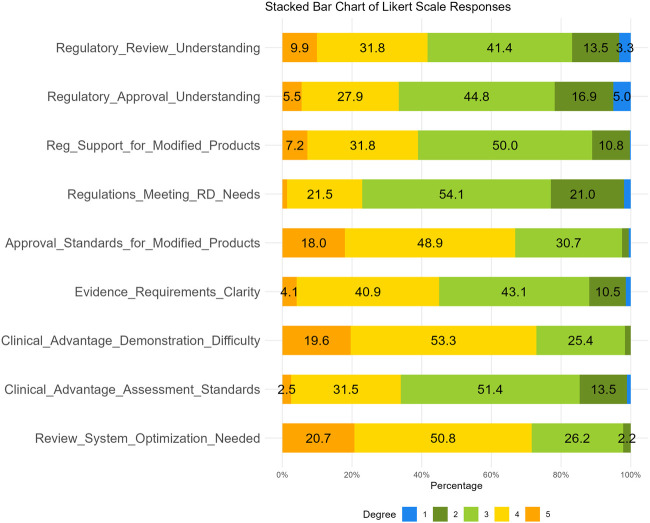
Responses to Likert Scale Items. ^a^ Likert scale ranges from 1 (Strongly Disagree) to 5 (Strongly Agree).

The fourth section consists of nine questions designed to assess the survey respondents’ understanding degree of drug regulatory laws and regulations. For this section, a Likert scale method was used, with each question offering five options ranging from 1 to 5, including “strongly disagree, disagree, neutral, agree, and strongly agree”. The results of these questions are illustrated in Table 7; Figure 8. This study found that about half of the respondents were either very familiar (9.9%) or familiar (31.8%) with drug regulation in China. However, only 5.5% were very familiar with the regulatory authorities’ approval attitudes and policy inclinations towards modified new products, while nearly a half (44.8%) held a neutral attitude. As for the current degree of support provided by regulations and policies for the development of modified new products, 39% agreed (including strongly agree) and 50% held a neutral attitude. Additionally, 22.9% of the respondents considered that existing regulations, policies, and technical guidelines cannot meet the industry’s needs for the development and registration of MNCDs. Notably, 66.9% of respondents (48.9% agreed and 18.0% strongly agreed) perceived the approval standards for MNCDs as high, and nearly 72.9% (53.3% agreed, 19.6% strongly agreed) thought it was difficult to demonstrate or assess the clinical advantages of MNCDs. Furthermore, 71.5% thought the regulatory review system for MNCDs requires further optimization and improvement.

**TABLE 7:** Responses to Likert scale items (N = 362).

Items	Strongly Disagree No. (%)	Disagree No. (%)	Neutral No. (%)	Agree No. (%)	Strongly Agree No. (%)	Mean ± SD
Regulatory Review Understanding	12 (3.3%)	49 (13.5%)	150 (41.4%)	115 (31.8%)	36 (9.9%)	3.3 ± 0.9
Regulatory Approval Understanding	18 (5.0%)	61 (16.9%)	162 (44.8%)	101 (27.9%)	20 (5.5%)	3.1 ± 0.9
Regulatory Support for Modified Products	1 (0.3%)	39 (10.8%)	181 (50.0%)	115 (31.8%)	26 (7.2%)	3.3 ± 0.8
Regulations Meeting RD Needs	7 (1.9%)	76 (21.0%)	196 (54.1%)	78 (21.5%)	5 (1.4%)	3.0 ± 0.7
Approval Standards for Modified Products	2 (0.6%)	7 (1.9%)	111 (30.7%)	177 (48.9%)	65 (18.0%)	3.8 ± 0.8
Evidence Requirements Clarity	5 (1.4%)	38 (10.5%)	156 (43.1%)	148 (40.9%)	15 (4.1%)	3.4 ± 0.8
Clinical Advantage Demonstration Difficulty	NA	6 (1.7%)	92 (25.4%)	193 (53.3%)	71 (19.6%)	3.9 ± 0.7
Clinical Advantage Assessment Standards	4 (1.1%)	49 (13.5%)	186 (51.4%)	114 (31.5%)	9 (2.5%)	3.2 ± 0.7
Review System Optimization Needed	NA	8 (2.2%)	95 (26.2%)	184 (50.8%)	75 (20.7%)	3.9 ± 0.7

^a^ Values are presented as n (%).

^b^ SD, standard deviation; Likert scale ranges from 1 (Strongly Disagree) to 5 (Strongly Agree).

At the end of the questionnaire, two open-ended questions were included to collect respondents’ suggestions on the evaluation standards for MNCD products, as well as on related aspects of R&D and regulation. The collected responses were categorized and statistically analyzed, with the results summarized in Table 8. The findings indicate that approximately half of the participants (50%) responded to these two open-ended questions. Among those who responded, 40% offered suggestions related to the clinical advantage evaluation standards for MNCDs. Additionally, some respondents highlighted the necessity to further refine the current regulatory standards. This could be achieved by providing more detailed technical guidelines and incorporating additional case references. Regarding the regulation of MNCDs, some respondents emphasized the importance of enhancing communication between industry stakeholders and regulators, while also advocating for more comprehensive guidance and practical examples.

**TABLE 8:** Summary of the suggestions for the regulation of MNCDs.

Suggestions	Suggestions, n (%) (N = 362)
Suggestions for the evaluation standards for MNCDs	
None	182 (50.3%)
Refine clinical advantage evaluation standards	74 (20.4%)
Others	21 (5.8%)
Clarify/optimize regulation requirements	14 (3.9%)
Clarify clinical advantages standards and clinical evidence requirements	11 (3.0%)
Add/refine relevant guidance documents	10 (2.8%)
Refine clinical advantages evaluation standards and guidance documents	9 (2.5%)
Optimize/simplify review and approval process	9 (2.5%)
Clarify/refine clinical evidence submission requirements	7 (1.9%)
Align with international standards	7 (1.9%)
Add case examples for guidance	7 (1.9%)
Refine clinical advantages evaluation standards and align with international standards	5 (1.4%)
Provide more guidance documents and case examples	4 (1.1%)
Clarify clinical advantages standards and provide more case examples	2 (0.6%)
Suggestions for R&D as well as regulation of MNCDs	
None	205 (56.6%)
Enhance communication and guidance	26 (7.2%)
Optimize review and approval processes and increase transparency	25 (6.9%)
Clarify/Improve regulation requirements	24 (6.6%)
Others	18 (5.0%)
Policy support	15 (4.1%)
Clarify regulation requirements and optimize review and approval processes	10 (2.8%)
Provide more guidance documents or case references	8 (2.2%)
Clarify/Improve regulation requirements and provide guidance documents	6 (1.7%)
Healthcare Insurance	6 (1.7%)
Align with international standards	6 (1.7%)
Clarify/Improve regulation requirements and align with international standards	4 (1.1%)
Refine clinical advantage evaluation standards and evidence requirements	4 (1.1%)
Optimize review and approval processes and provide more guidance documents	3 (0.8%)
Increase industry consensus	2 (0.6%)

## 4 Discussion

Since the drug regulation reform, the new concept of modified new drug has emerged in China, used for non-innovative and non-generic drug registration and regulation. Actually, this concept is similar to the 505(b) (State Administration for Market Regulation, 2020) NDA pathway in the United States and the hybrid application pathway in Europe in the area of drug registration. A 505(b) (State Administration for Market Regulation, 2020) application is an NDA that includes comprehensive safety and effectiveness investigation reports, in which at least some of the necessary information for approval is derived from studies not conducted by or for the applicant, and for which the applicant has not obtained the right of reference or use (U.S. Food and Drug Administration, 2019; United States Congress, 2024). A hybrid application means the drug product for marketing authorization does not fall within the definition of a generic medicinal product or where the bioequivalence cannot be demonstrated through bioavailability studies. This includes the case of changes in the active substance(s), therapeutic indications, strength, pharmaceutical form or route of administration, compared with the reference medicinal product (European Union, 2022). Compared to the concept of the 505(b) (State Administration for Market Regulation, 2020) NDA or the hybrid application pathway, the concept of modified new drug is relatively new, which has been implemented for only 8 years.

To the best of our knowledge, this survey was the first study conducted to assess the current status of MNDCs in China. The lack of similar studies prior to this highlights the novelty and critical importance of our research in addressing this significant knowledge gap. The scarcity of existing research may be attributed to three key factors: 1. the emerging regulatory framework established within the past decade; 2. the conceptual complexity of modified new drugs, encompassing a range of modifications from structural changes to new indications; 3. the recent emergence and development of regulatory science in China.

With the deepening of drug reform and the optimization of the current regulatory review and approval system for MNCDs, conducting such a survey is crucial. By providing the first empirical insights into the current status of modified new drug regulations, our study offers a pioneering perspective that may guide future research and policy development in pharmaceutical innovation. The results of this research can reflect the industry’s general viewpoints on MNCDs regulation. The survey identified the critical issues and challenges during the R&D and regulatory submission processes of MNCDs. Additionally, the survey results provide a comprehensive overview related to the industry perspective about the MNCDs regulation in China, which could provide insights for the refinement of MNCDs regulation. Firstly, the respondents are affiliated with over 178 institutions across 19 provinces in China, and 90.3% of respondents have experience in R&D of MNCDs. In addition, 85% of the respondents, affiliated with 178 primary affiliations are actively developing MNCDs, and another 5.2% are planning to develop MNCDs. This indicates that the respondents and their affiliations come from various regions across the country, reflecting a broad and representative sample of professionals in the field of modified new drugs. Secondly, the highest R&D stage of the MNCDs in their affiliations span all phases, MNCD types and therapeutic indications, and 96% of respondents hold an optimistic opinion on the prospects of MNCDs. Furthermore, the number of IND approvals rapidly increased during the period from 2017 to 2024 (Figure 1). These results suggest that the industry actively participates in the R&D of MNCDs. This also reflects the current state of modified new drugs and indicates that the prospects for such drugs are very broad. Thirdly, our findings suggest that the main issue encountered in the process of regulatory submission of MNCDs is the lack of clear guidelines and case references, and the main challenge is the evaluation of clinical advantages of MNCDs. Actually, the guidance issued by CDE has illustrated the principle of demonstrating the clinical advantage of MNCDs, but the study results still reflect this as the main regulatory challenge. The reason for this may be due to the fact that the guidance is not sufficiently objective and clear, or there is inconsistency and imbalance in the views on the clinical advantages of modified new drugs between reviewers and stakeholders. The establishment of guidance should also be periodically updated and revised. This indicates an urgent need for an updated and more objective standard for assessing clinical advantages of modified new drugs to assist their development.

Additionally, the respondents identified clinical advantage as the most critical focus in the regulatory review process for modified new products. Respondents indicated that there is currently a lack of a standardized assessment system for evaluating the significant clinical advantages of MNCDs, as well as insufficient clarity regarding the degree of these advantages. The factor that affects the market launch of MNCDs most is clinical trial efficacy and safety data, and the most common approach used to assess the clinical advantages of MNCDs is to consult experts. Obviously, there is an urgent need for clearer guidance and more scientific methods to assist the industry in assessing the clinical advantages of MNCDs. We traced the regulatory policies, regulations and guidance related to modified new drugs since the drug review and approval reform in 2015. We found that the requirement for clinical advantages was initially mentioned in the “*Announcement on Certain Policies Regarding Drug Registration, Review and Approval*” (No. 230 of 2015) issued by the China Food and Drug Administration (CFDA, the former of the NMPA) in November 2015 (China Food and Drug Administration, 2015). The original text stated: “For drug registration applications that change the original drug’s dosage form, acid radical, base, or administration route, applicants need to prove their technical innovation and demonstrate significant clinical advantages compared to the original product; those unable to prove such advantages will not be approved. This excludes pediatric drug applications for changes in dosage form and specifications.” Subsequently, including the “*Work Plan for Chemical Drug Registration Classification Reform*” issued in 2016 (China Food and Drug Administration, 2016) and the “*Classification and Submission Requirements for Chemical Drugs*” issued in 2020 (National Medical Products Administration, 2020a), all required modified new drugs to demonstrate significant clinical advantages.

Until December 2020, the definition of the clinical advantages of modified new drugs was clearly outlined in the “*Technical Guidance for Clinical Trials of Modified New Chemical Drugs*” issued by CDE of the NMPA (National Medical Products Administration, 2020b). This definition emphasizes the need to address unmet clinical needs for patients, and requires that modified new drugs should demonstrate substantial improvements in efficacy, safety, or compliance compared to existing medications. This requirement suggests that drugs lacking substantial clinical advantages may face approval challenges. The feedback from the respondents in the survey questionnaire clearly reflects the current biggest challenge for modified new drugs in obtaining regulatory approval: how to effectively demonstrate their clinical advantages over existing therapies. These survey results also raise several new questions for both the industry and regulators to consider: How are “significant clinical advantages” evaluated by regulators? Are there specific assessment systems or standards in place within the drug regulatory authorization process? How should developers of modified new drugs conduct self-assessment of their product’s clinical advantages to meet regulatory requirements and standards? It appears that these questions currently lack definitive answers and are worthy of further in-depth research in the future. Regulatory science involves developing new tools, standards, and methods to assess the safety, efficacy, quality, and performance of regulated products (U.S. Food and Drug Administration, 2011). Here, regulatory science methods can be employed to study new standards, methods, and tools to support the assessment of clinical advantages for modified new drugs.

In addition to safety, efficacy, and compliance improvement, we further investigated the respondents’ other additional considerations regarding clinical advantages. The results indicated that other dimensions that may need to be taken into account include but not limited to technological innovation, economic viability, convenience, accessibility, and special population needs. For the further balance between industry and regulators, we also designed several questions in the third section of the survey involving some suggestions for the regulation of MNCDs. The findings indicated that there are several aspects that can be considered in the refinement of drug regulatory review process of MNCDs, which include enhancing the transparency and consistency of review criteria, providing more guidance, case references, and detailed feedback and recommendations for the industry.

## 5 Conclusion

Modified new drugs are improvements to existing medications, aimed at improving access to better medications and enhancing the patient experience. This study presents a comprehensive landscape of stakeholders’ current perspectives on the regulation of MNCDs. According to the insights garnered from this survey, the development and approval process of MNCDs face significant challenges, and the most critical challenge encountered by MNCD developers in China is how to assess and demonstrate the significant clinical advantage. There is an urgent industry call for more definitive guidance and aligned regulatory policies. Consequently, a common and shared assessment system needs to be studied to evaluate the clinical advantages of MNCDs, and balance the expectations between regulators and the industry. Furthermore, the survey also showcases substantial potential and identified additional dimensions that could be considered to facilitate the R&D of MNCDs. Ongoing communication between regulatory agencies and the pharmaceutical industry is crucial to advance the development of MNCDs and ensure patients’ access to safe, effective, and affordable medications. Overall, this study’s findings offer value and reference for all domestic and international drug R&D professionals, stakeholders, and policymakers, especially for the regulators to optimize the clinical advantage assessment system for MNCDs.

## Data Availability

The raw data supporting the conclusions of this article will be made available by the authors, without undue reservation.
