# Supplementary material for: Key regulatory challenges in developing modified new chemical drugs in China: a national survey study
Source: Front Pharmacol. 2025 Jun 3;16:1576013. doi: 10.3389/fphar.2025.1576013 (PMC12170327; doi:10.3389/fphar.2025.1576013)
Supplement: Supplementary file 1 [file Supplementaryfile1.docx]

**Content of Survey Sent to participants**

**Cover letter**

Since the implementation of drug regulation reforms in China in 2015, a series of policies have been introduced to encourage the development of new drugs, along with a readjustment of the drug registration classification system. Among them, the registration classification of chemical drugs is categorized into innovative drugs, modified new drugs, and generic drugs. Modified new chemical drugs refer to the optimization of the structure, dosage form, formulation process, route of administration, indications, etc., based on known active ingredients, resulting in drugs with significant clinical advantages. Compared to innovative drugs and generic drugs, modified new drugs are characterized by low risk, short development cycles, high success rates, and high technical barriers. These advantages have increasingly made modified new drugs a hotspot in drug research and development (R&D), attracting more and more companies to invest in their development.

However, modified new drugs in China face numerous challenges throughout the R&D and marketing authorization process, including issues related to technological innovation, market competition, and regulatory oversight. Considering this situation, we have initiated a national survey from the perspective of regulatory science, to understand and identify the key regulatory issues in the R&D and marketing authorization process of modified new drugs. This survey aims to explore new standards, new tools, and new methods for the regulation of modified new drugs. The findings of the survey will provide valuable decision support for policymakers, pharmaceutical companies, and regulatory agencies, ultimately promoting more efficient review and regulation for modified new drugs.

Note: In this questionnaire, the term “modified new drugs” specifically refer to modified new chemical drugs as mentioned in the following text.

**Confidentiality Measures**

Thank you for reading and completing this anonymous survey questionnaire. This survey is conducted solely for academic research purposes, and we committed to keep the information you provide confidential. Your opinions and suggestions are vital to our research work.

**1. Information Security Confirmation**

By completing this research questionnaire, you consent to the organizer’s collection, reproduction, and use of the information you voluntarily provide. We will adhere to industry standards for information security management to protect sensitive information. We will only retain your information as permitted by law and, in any case, only for the duration allowed by law.

[Single Choice] *

| ○Yes |
| --- |

This questionnaire consists of 40 questions and is expected to take you 5-10 minutes to read and answer.

# Section 1 General Background Information

2. Gender： [Single Choice] *

| ○Male | ○Female |
| --- | --- |

3. Age： [Single Choice] *

| ○Under 25 | ○25-34 |
| --- | --- |
| ○35-44 | ○45-54 |
| ○55-64 | ○65 and over 65 |

4. What is your highest degree: [Single Choice] *

| ○Below undergraduate | ○Undergraduate |
| --- | --- |
| ○Master | ○PhD |
| ○Others: _________________ |  |

5. How many years have you been working in the pharmaceutical industry: [Single Choice] *

| ○less than 1 year | ○1-5years |
| --- | --- |
| ○5-10 years | ○10-15 years |
| ○over 15 years | ○Others: _________________ * |

6. Your position/professional title: [Single Choice] *

| ○Senior |
| --- |
| ○Mid-Senior |
| ○Intermediate |
| ○Junior |
| ○Others: _________________ * |

7. The area of specialization in which you are currently working is: [Multiple Choice] *

| □Basic scientific research | □Formulation development | □Non-clinical study |
| --- | --- | --- |
| □Clinical study | □Drug registration | □Government affairs work |
| □Post-marketing research | □Health Technology Assessment | □Others: _________________* |

8. The name of your employment sector：[Fill-in-the-blank] *

_________________________________

9. What type of institution does your affiliation belong to: [Multiple Choice] *

| □Pharmaceutical R&D companies | □Pharmaceutical manufacturing companies |
| --- | --- |
| □Universities or research institutes | □Contract Research Organizations (CRO companies) |
| □Contract Development and Manufacturing Organizations (CDMO companies) | □Hospital/Clinic |
| □Others: _________________ | |

10. The nature of your organization is: [Single Choice] *

| ○Domestic-funded enterprise | ○Foreign-funded enterprise |
| --- | --- |
| ○Sino-foreign joint venture | ○Public institution |
| ○Others: _________________ | |

11. The city where you are currently working is: [[Fill-in-the-blank: Province - City] *

_________________________________

12. The main product areas that your organization focuses on or is involved in research and development: [Multiple Choice] *

| □Chemical drugs | □Biological products |
| --- | --- |
| □Traditional Chinese medicine | □Medical devices |
| □Health and wellness sector | □Others: _________________* |

13. Is your organization currently working on or planning to develop modified new drugs? [Single Choice] *

| ○Yes |
| --- |
| ○No **(Please skip to question 17)** |
| ○Planning |

14. What is the highest stage of development for the modified new drugs in your organization: [Single Choice] *

| ○Technology Development / Academic Research | ○Project Initiation Phase | ○Pharmaceutical Research (Formulation and Process Development) |
| --- | --- | --- |
| ○Preclinical Research | ○IND or Filing | ○Phase 1 Clinical Study |
| ○Phase 2 Clinical Study | ○Phase 3 Clinical Study | ○Marketing Application Phase |
| ○Post-marketing Research | | |

15. Types of modified new drugs your organization is developing or focusing on: [Multiple Choice] *

| □New Active Ingredients | □New Dosage Forms |
| --- | --- |
| □New Fixed-Dose Combinations | □New Indications |
| □Others: _________________* | |

16. What are the indications for the modified new products that your organization is focusing on or developing? [Multiple Choice] *

| □A Alimentary Tract and Metabolism |
| --- |
| □B Blood and Blood Forming Organs |
| □C Cardiovascular System |
| □D Dermatological |
| □G Genito Urinary System and Sex Hormones |
| □H Systemic Hormonal Preparations, Excl. Sex Hormones and Insulins |
| □J Anti-infective for Systemic Use |
| □L Antineoplastic and Immunomodulating Agents |
| □M Musculo-Skeletal System |
| □N Nervous System |
| □P Antiparasitic Products, Insecticides and Repellents |
| □R Respiratory System |
| □S Sensory Organs |
| □V Various |

# Section 2 Key Issues and Challenges in the development and Market Launch of Modified New Drugs

17. Which of the following types of experience do you have in the R&D or regulatory submission of modified new drug products? [at least 1 choice(s)] *

| □Experience in optimizing the structure of known active ingredients (such as splitting or synthesizing optical isomers, esterification, salt formation, changing acid radicals, bases, or metal elements, forming non-covalent derivatives, etc.) |
| --- |
| □Experience in developing new dosage forms (including new drug delivery systems), new formulation processes, and new routes of administration |
| □Experience in developing new fixed-dose combinations |
| □Experience in developing new drug indications |
| □Experience in regulatory submission related to modified new drugs |
| □Experience in non-clinical studies of modified new drugs |
| □Experience in clinical studies of modified new drugs |
| □No experience in any of the above |
| □Others: __________________________________* |

18. What are the main issues you encounter during the drug registration and submission process?

[Ranking question, please fill in the numbers in the brackets in order] *

| [ ] Complex registration documentation requirements |
| --- |
| [ ] Long review times and slow feedback |
| [ ] Poor communication with regulatory agencies |
| [ ] Frequent changes in regulations, difficult to keep up |
| [ ] Lack of clear guidelines and case references |
| [ ] Others: __________________________________* |

19. What do you consider the main challenges currently faced in the R&D and market application of modified new drugs?

[Ranking question, please fill in the numbers in the brackets in order] *

| [ ] Basis for project initiation |
| --- |
| [ ] Assessment and proof of clinical advantages |
| [ ] Requirements for evidence supporting market launch |
| [ ] Breaking through technical barriers and innovation |
| [ ] Design of clinical trials |
| [ ] Others: __________________________________* |

20. What factors do you think have the greatest impact on the successful market launch of modified new drugs?

[Ranking question, please fill in the numbers in the brackets in order] *

| [ ] Quality and depth of preclinical research |
| --- |
| [ ] Efficacy and safety data from clinical trials |
| [ ] Regulatory compliance and registration strategy |
| [ ] Market analysis and positioning |
| [ ] Ongoing investment of funds and resources |
| [ ] Others: __________________________________* |

21. During the development phase of modified new drug products, which approach does your organization most commonly use to assess clinical advantages?

[Ranking question, please fill in the numbers in the brackets in order] *

| [ ] Consulting experts |
| --- |
| [ ] Literature research |
| [ ] Scientific research |
| [ ] Communication with regulatory agencies |
| [ ] Market research and demand analysis |
| [ ] Others: ___________________________________* |

22. The regulatory requirements for clinical advantage assessment refer to effectiveness, safety, and compliance. What other dimensions do you think regulatory review should also consider? [Multiple Choice] *

| □Technological innovation | □Economic viability |
| --- | --- |
| □Convenience | □Accessibility |
| □Special populations (such as rare diseases) | □Others: _________________* |

23. What is your opinion on the prospects of modified new drugs? [Single Choice] *

| ○There is still a large amount of unmet clinical needs, with broad prospects |
| --- |
| ○There are specific clinical needs, with a certain market space |
| ○Clinical needs are basically met, and the market is close to saturation |
| ○Uncertain |

# Section 3 Regulatory Challenges in the Registration Application of Modified New Drugs

24. Which policies or regulations do you think have the greatest impact on the R&D of modified new drugs? [Single Choice] *

| ○Drug registration and review policies |
| --- |
| ○Regulations related to clinical trials |
| ○Intellectual property protection and data exclusivity |
| ○Drug safety and quality standards |
| ○Market access and healthcare insurance policies |
| ○Others: ___________________________________* |

25. What do you think is the most important focus in the regulatory review process for modified new drugs? [Single Choice] *

| ○Clinical advantage |
| --- |
| ○Clinical evidence |
| ○Non-clinical evidence |
| ○Formulation process |
| ○Quality control |
| ○Others: ___________________________________* |

26. What do you think is the most area that regulatory agencies need to improve in the review process of modified new drugs? [Single Choice] *

| ○Enhancing the transparency and consistency of review criteria |
| --- |
| ○Strengthening professional training and capacity building of reviewers |
| ○Optimizing review processes and time management |
| ○Improving communication and feedback mechanisms with enterprise |
| ○Providing more guidelines and case references |
| ○Others: ___________________________________* |

27. What suggestions do you have for improving the transparency and efficiency of the review process for modified new products? [Multiple Choice] *

| □Make more review report information public |
| --- |
| □Provide more detailed review feedback and guidance |
| □Establish more efficient communication channels |
| □Increase reviewers and resources |
| □Introduce external experts and consultants |
| □Others: ___________________________________* |

28. Regarding the construction and improvement of the regulatory system and technical standards for modified new drug products in our country, which aspects do you think should be addressed? [Multiple Choice] *

| □Laws |
| --- |
| □Administrative regulations |
| □Technical guidelines |
| □Expert consensus |
| □Industry standards |
| □Others: ___________________________________* |

# Section 4 Degree of Understanding of Drug Regulatory Laws and Regulations

29. Your perceived level of familiarity with drug regulation: [Single Choice] *

| ○Not at all familiar | ○Not very familiar | ○Neutral | ○Fairly familiar | ○Very familiar |
| --- | --- | --- | --- | --- |

30. Your perceived level of familiarity with the regulatory authorities’ approval attitudes and policy orientations towards modified new drug products? [Single Choice] *

| ○Not at all familiar | ○Not very familiar | ○Neutral | ○Fairly familiar | ○Very familiar |
| --- | --- | --- | --- | --- |

31. How do you rate the current degree of support from regulations and policies for the development of modified new drug products? [Single Choice] *

| ○Very unsupportive | ○Not very supportive | ○Neutral | ○Comparatively supportive | ○Very supportive |
| --- | --- | --- | --- | --- |

32. Do you think the existing regulations, policies, and technical guidelines can meet the industry needs for the development and registration of modified new products? [Single Choice] *

| ○Very unsatisfied | ○Unsatisfied | ○Neutral | ○Satisfied | ○Very satisfied |
| --- | --- | --- | --- | --- |

33. What is your opinion on the current regulatory agencies’ review standards and requirements for approving modified new drug products? [Single Choice] *

| ○Much too low | ○Too low | ○Neutral | ○Too high | ○Much too high |
| --- | --- | --- | --- | --- |

34. Do you think the current regulatory agencies’ requirements for evidence supporting the development and market launch of modified new drug products are clear? [Single Choice] *

| ○Very unclear | ○Not very clear | ○Neutral | ○Fairly clear | ○Very clear |
| --- | --- | --- | --- | --- |

35. Regarding the current regulatory requirement that modified new drug products should have a clear clinical advantage, how difficult do you think it is to prove or assess this clinical advantage? [Single Choice] *

| ○Very easy | ○Fairly easy | ○Neutral | ○Fairly difficult | ○Very difficult |
| --- | --- | --- | --- | --- |

36. Do the current review technical standards meet the assessment of the clinical advantages of modified new drug products？[Single Choice] *

| ○Not at all satisfied | ○Not satisfied | ○Neutral | ○Satisfied | ○Very satisfied |
| --- | --- | --- | --- | --- |

37. Do you think the current regulatory review system for modified new drug products needs further optimization and improvement? [Single Choice] *

| ○Not at all needed | ○Not very needed | ○Neutral | ○Fairly needed | ○Very needed |
| --- | --- | --- | --- | --- |

38. What are your suggestions for improving the evaluation standards for domestic modified new drug products (such as standards for assessing clinical advantages)? [Fill-in-the-blank] *

_________________________________________________________________________________

39. Do you have any other opinions or suggestions regarding the R&D, registration, and regulatory review of modified new drug products？[Fill-in-the-blank] *

_________________________________________________________________________________

40. **Closing Remarks**

Thank you for your participation and valuable feedback.

If you would like to receive the results of the follow-up survey, please leave your email address [Fill-in-the-blank]:

__________________________________________________________________
